# Depression, anxiety, stress symptoms and their determinants among secondary students with vision impairment in rural Northwestern China during the COVID-19 pandemic

**DOI:** 10.3389/fpubh.2023.1282826

**Published:** 2024-01-24

**Authors:** Dongfeng Li, Ving Fai Chan, Huan Wang, Huiping Zhang, Gianni Virgili, Noelle Whitestone, Baixiang Xiao, Manpreet K. Singh, Xinshu She, Graeme Mackenzie, Matthew Boswell, Sonia Mavi, Scott Rozelle, Nathan Congdon

**Affiliations:** ^1^Department of Ophthalmology, Sichuan Provincial People’s Hospital, University of Electronic Science and Technology of China, Chengdu, China; ^2^Chinese Academy of Sciences Sichuan Translational Medicine Research Hospital, Chengdu, China; ^3^Centre for Public Health, Queen’s University Belfast, Belfast, United Kingdom; ^4^Stanford Centre on China’s Economy and Institutions, Stanford University, Stanford, CA, United States; ^5^School of Financial and Management, Ningxia University, Yinchuan, China; ^6^Department NEUROFARBA, University of Florence, Florence, Italy; ^7^Orbis International, New York, NY, United States; ^8^Affiliated Eye Hospital of Nanchang University, Nanchang, China; ^9^Stanford University School of Medicine, Stanford University, Stanford, CA, United States; ^10^Clearly Initiatives, London, United Kingdom; ^11^Zhongshan Ophthalmic Centre, Sun Yat-sen University, Guangzhou, China

**Keywords:** mental health, children, vision impairment, rural China, COVID-19

## Abstract

**Objective:**

The measures implemented to control the spread of Coronavirus disease 2019 (COVID-19) could affect children’s mental and vision health. Youth particularly from minority and socioeconomically disadvantaged backgrounds were more likely to be impacted by these measures. This study aimed to examine the mental health of children with vision impairment and associated factors in North-western China during the COVID-19 pandemic.

**Methods:**

A cross-sectional study was conducted among 2,036 secondary school children living in Ningxia Hui Autonomous Region. Participants completed a survey on sociodemographic and lifestyle information and answered the Chinese version of the 21-item Depression Anxiety Stress Scales (DASS-21) questionnaire. Presenting visual acuity was measured by a trained enumerator. Multivariate logistic regression analysis was used to identify potential risk factors for mental health problems.

**Results:**

Responses from 1,992 (97.8%) children were included in the analysis after excluding those with incomplete mental health outcome data. The prevalence of depression, anxiety and stress symptoms within the dataset were 28.9, 46.4, and 22.3%, respectively. The distribution of children with different stress levels differed significantly between those with and without vision impairment (*p* = 0.03). Multivariable logistic regression analyses revealed that depression symptoms decreased with higher parental education (OR, 0.76, 95% confidence intervals (CI):0.63–0.96), longer sleep duration (OR, 0.90, 95% CI: 0.81–0.97) and longer study time (OR, 0.82, 95% CI: 0.74–0.91), whereas they increased with higher recreational screen time (OR, 1.19, 95% CI: 1.08–1.32). Anxiety symptoms decreased with higher parental education (OR, 0.80, 95% CI: 0.66–0.96) and increased with higher recreational screen time (OR, 1.15, 95% CI: 1.04–1.27) and being a left-behind child (OR, 1.26, 95% CI: 1.04–1.54). In addition, stress symptoms decreased with longer sleep duration (OR, 0.92, 95%CI: 0.85–0.99) and increased with higher number of siblings (OR, 1.10, 95% CI: 1.01–1.19), higher recreational screen time (OR, 1.15, 95% CI: 1.04–1.28) and older age (OR,1.12, 95% CI: 1.004–1.24).

**Conclusion:**

A considerable proportion of our sample experienced mental health problems during the pandemic. Healthcare planners in China should consider interventions such as reducing recreational screen time, ensuring sufficient sleep, and timely detection of mental health symptoms among socioeconomically disadvantaged groups.

## Introduction

In December 2019, a novel Coronavirus pandemic rapidly spread worldwide. Governments implemented various measures to control the pandemic on an international scale, such as restrictions on public activities, home quarantine, physical distancing, and school closures. Strict pandemic prevention measures led to a decrease in time spent outdoors and an increase in screen time (1, 2), which may have detrimentally impacted individuals’ vision and mental health during the COVID-19 pandemic (3–6).

Globally, one in seven 10 to 19-year-old children and young adults experience a mental disorder, and depression and anxiety disorders are among the leading causes of illness and disability among adolescents (7). When COVID-19 was declared an international public health emergency, children worldwide experienced dramatic disruptions to their daily lives, and the prevalence of depression and anxiety increased correspondingly. A systematic review of the global literature comprising 29 articles found that the prevalence of clinically-elevated depression and anxiety in children doubled compared with the pre-pandemic period, reaching 25.2 and 20.5%, respectively (8).

Ningxia is an autonomous region located in Northwest China with a population of 6.62 million, of which 38% are Hui ethnicity (9). Ningxia is a relatively undeveloped area, its disposable income per capita in 2020 was approximately RMB 27904 (US$3986), ranking 20th among 32 administrative regions in China (10). Ningxia reported the first COVID-19 cases in Jan 2020 (11), and all primary and secondary students in Ningxia were confined at home for almost 4 months with online school lessons (12). This led to increased screen time and reduced outdoor time, both of which have been shown to have a detrimental effect on children’s mental health (13, 14). Home confinement during the COVID-19 pandemic also increased the incidence and progression of myopia (5), which is the leading cause of children’s vision impairment and has also been shown to impact children’s mental health negatively (15).

These studies have significant implications for China as (i) vision impairment and myopia are among the most common health problems in Chinese students (16, 17); (ii) preliminary evidence also suggests an increased incidence and progression of myopia among children during the COVID-19 outbreak in China (1, 5); and (iii) the combination of the lockdown during the COVID-19 pandemic, uncorrected myopia, and subsequent unemployment and income reduction (18) may disproportionately affect the mental health of children from low socioeconomic status families (19). However, it is unclear how children’s mental health in rural China, especially rural minority populations, was affected by quarantine measures during COVID-19 and its association with the aforementioned factors. The Hui community, predominantly adherents of the Muslim faith, possesses a distinctive culture. They adhere to Islamic dietary laws and actively participate in various religious activities (20). In the rural part of Ningxia the Hui community is less educated and has a lower household per capita wealth compared to the Han community in the same region (21). Furthermore, given China’s relatively permissive birth-control policy for ethnic minorities, Hui households tend to have a higher number of dependent children compared to their Han counterparts (22). All these factors may contribute to the poor mental health of Hui people. Studies from Ningxia found that high religiosity was associated positively with mental disorders in older Hui people (23). Studies before COVID-19 indicated that adults women of Hui ethnicity in rural Ningxia experience more severe mental health symptoms including depression and anxiety than their Han counterparts (24). Children of Hui ethnicity from the Ningxia region are particularly vulnerable to behavioral and emotional problems (25, 26). Few studies have focused on investigating the mental health of children from the Hui ethnicity and other minority groups during the COVID-19 pandemic. Further, it is unclear how Hui children were affected psychologically during the pandemic.

Thus, this study aimed to assess the mental health of Hui and Han children in rural Northwestern China using the Depression Anxiety Stress Scales (DASS-21) and to explore associations among mental health, vision impairment, and socioeconomic and lifestyle factors.

## Methods

The protocol for this study was approved by the Institutional Review Boards at Stanford University (Protocol ID 52514) and Queen’s University Belfast (PFREC 19.25). Written informed consent was obtained from the parents of the students before data collection began. The study adhered to the tenets of the Declaration of Helsinki.

### Study design and participants

This cross-sectional study was conducted in October 2021. The study was part of the See Well to stay In ScHool (SWISH) trial, designed to assess whether glasses distribution to myopic students in secondary school can increase academic high school attendance rates and improve mental health status in rural areas (27). As per the SWISH protocol, we aimed to screen 20,000 students of grade 7 and 8 from 120 secondary schools in Ningxia autonomous region. However, due to the outbreak of COVID-19 in Ningxia in October 2021, the included schools were temporarily closed. Fieldwork was suspended after 2,036 children from 13 schools in three counties were screened. After excluding participants with incomplete mental health data (*n* = 44), 1,992 participants (97.8%) were included in the analysis.

### Measures

Field teams of 10 trained enumerators recruited from a local university administered a questionnaire to the children to collect data on sociodemographic status. Information was sought on age, gender, parental marital status, and whether or not they were a “left-behind child” [defined as children who live in their rural communities while one or both of their parents have migrated into cities for work over 6 months (28)], parental education level, and family wealth. In addition, questions were asked regarding lifestyle habits, including self-reported sleep time, study time, recreational screen time, and time spent on outdoor activities. Family wealth was measured by a pre-defined list of assets, including ownership of a private car, an apartment in the county, a refrigerator at home etc. We constructed a family wealth index by combining the self-reported assets using Principal Component Analysis (PCA) (29). The wealth index value was categorized in terciles and, used in the regression analysis. The lowest tercile was classified as poor, the highest was rich, and the rest were of average economic status.

Students were administered the Depression Anxiety Stress Scale (DASS-21) to determine their mental health (30). The DASS-21 is a self-reported measure of negative effects with three 7-item subscales (depression, anxiety, and stress). The responses were indicated using a 4-point Likert scale ranging from 0 (“does not apply to me at all”) to 3 (“applies to me very much or most of the time”), with higher scores indicating more negative experiences in the past week. Scores for each subscale were obtained by totaling the responses to the component items. DASS-21 has been previously used to assess mental health in Chinese populations (31).

After completing the questionnaires, the student’s visual acuity was assessed. The process has been described in detail by Wang et al. (32), and briefly included testing presenting visual acuity one eye at a time at a distance of 4 meters using the Early Treatment Diabetic Retinopathy tumbling E study chart (Precision Vision, La Salle, IL) in a well-lit, indoor area. Children with glasses were asked to wear them during the assessment. Visual acuity was recorded using logMAR (log of the Minimum Angle of Resolution) values. Presenting vision impairment was defined as better-eye presenting vision >0.3 logMAR.

### Statistical analysis

All statistical analyses were performed using Stata 17.0 (Stata Corp., College Station, TX). Descriptive statistics were reported for the demographic data and prevalence of depression, anxiety, and stress symptoms among the participants. Associations between potential determinants and the symptoms of depression, anxiety and stress were investigated using univariable and multivariable logistic regression models. Odds ratios and 95% confidence intervals (CI) were also calculated. All variables with a *p* < 0.2 in the univariable logistic regression were included in the multivariable model. A two-tailed *p* < 0.05 was considered statistically significant.

## Results

The study included 1,992 children (49.3% boys, with a mean age of 13.5 ± 0.88 years). Children from the Hui ethnic group formed 77.5% of the sample (*n* = 1,541), while the rest were from the majority Han group. About a third of the sample (*n* = 608, 30.5%) were left-behind children. Most children (*n* = 1,044, 52.4%) had at least one parent with 9 years or more education, and 1,002 (50.3%) were boarding at school.

Children spent an average of 0.88 ± 0.96 h per day on recreational screen time (including television, smartphones, laptops, and tablets). The average outdoor activities time, sleep time and study time after school were 0.91 ± 0.69 h, 9.65 ± 1.19 h and 1.15 ± 1.09 h per day, respectively. About a fifth of participants (20.3, 95%CI, 18.6–22.1%) had vision impairment (Table 1).

**Table 1:** Demographic and lifestyle characteristics of participants (*N* = 1,992) in a study of mental health in rural Chinese school-children.

Variable	Total (mean ± SD or %)
Age (year)	13.5 ± 0.88
Grade	
7	1,044 (52.4%)
8	948 (47.6%)
Gender	
Female	1,009 (50.7%)
Male	983 (49.3%)
Family wealth index	
Lowest (Index value ≤33.3%)	814 (40.9%)
Middle (33.3% < Index value≤66.6%)	510 (25.6%)
Highest (Index value>66.6%)	668 (33.5%)
Ethnicity^*^	
Han	446 (22.5)
Hui	1,541 (77.5)
Visually impaired (vision in the better-seeing eye >0.3 logMAR)	
No	1,587 (79.7)
Yes	405 (20.3)
Boarding at school^**^	
Yes	1,002 (51.1)
No	959 (48.9)
Left behind children^***^	
Yes	608 (31.5)
No	1,320 (68.5)
Number of siblings	1.94 ± 1.19
Parents’ marital status	
Married	1793 (90.0)
Single	199 (10.0)
Parents’ education	
Both parents have <9 years of education	893 (46.1)
At least one parent has >9 years of education	1,044 (53.9)
Average study time per day (hour)	1.15 ± 1.09
Average screen time per day (hour)	0.88 ± 0.96
Outdoor activity time per day (hour)	0.91 ± 0.69
Sleep time per day (hour)	9.65 ± 1.19

*Six missing values; **31 missing values; ***64 missing values.

The prevalence of symptoms of depression was 28.9% (95% CI, 26.3–30.2%), while the figures for anxiety and stress symptoms were 46.4% (95% CI, 44.2–48.6%) and 22.3% (95%CI, 20.5–24.2%) respectively. The prevalence of extremely severe stress symptoms was significantly higher in children with vision impairment than those with normal vision. However, no significant differences were observed in the level of depression and anxiety symptoms among children with or without vision impairment. Ethnicity was not associated with the prevalence of depression, anxiety, and stress symptoms (Table 2).

**Table 2:** Prevalence of depression, anxiety and stress symptoms among rural students in China on the DASS-21, stratified by vision impairment and ethnicity.

Symptom	Categories	Total (%)	Vision status		Ethnicity	
			Normal vision	Vision impaired	Hui	Han
Depression	Between-group comparison (*p*-value)		*p* = 0.1		*p* = 0.16	
	No depression (0–9)	1,429 (71.1)	1,138 (71.7)	291 (71.8)	1,103 (71.6)	322 (72.2)
	Mild (9–13)	196 (9.84)	162 (10.2)	34 (8.40)	154 (9.99)	41 (9.19)
	Moderate (14–20)	224 (11.2)	179 (11.3)	45 (11.1)	174 (11.3)	50 (11.2)
	Severe (21–27)	87 (4.37)	71 (4.47)	16 (3.95)	73 (4.74)	14 (3.14)
	Extremely severe (≥28)	56 (2.81)	37 (2.33)	19 (4.69)	37 (2.40)	19 (4.26)
Anxiety	Between-group comparison (*p*-value)		*p* = 0.78		*p* = 0.99	
	No anxiety (0–7)	1,067 (53.6)	850 (53.6)	217 (53.6)	822 (53.3)	241 (54.0)
	Mild (8–9)	189 (9.49)	145 (9.14)	44 (10.9)	147 (9.54)	42 (9.42)
	Moderate (10–14)	389 (19.5)	314 (19.8)	75 (18.5)	301 (19.5)	88 (19.7)
	Severe (15–18)	163 (8.18)	133 (8.38)	30 (7.41)	127 (8.24)	35 (7.85)
	Extremely severe (≥19)	184 (9.24)	145 (9.14)	39 (9.63)	144 (9.34)	40 (8.97)
Stress	Between-group comparison (*P*-value)		*P* = 0.03		*p* = 0.79	
	No stress (0–14)	1,547 (77.7)	1,241 (78.2)	306 (75.6)	1,191 (77.3)	352 (78.9)
	Mild (15–18)	190 (9.54)	154 (9.70)	36 (8.89)	151 (9.80)	39 (8.74)
	Moderate (19–25)	155 (7.78)	123 (7.75)	32 (7.90)	122 (7.92)	32 (7.17)
	Severe (26–32)	75 (3.77)	55 (3.47)	20 (4.94)	56 (3.63)	19 (4.26)
	Extremely severe (33–42)	25 (1.26)	14 (0.88)	11 (2.72)	21 (1.36)	4 (0.90)

Multivariate logistic regression analysis revealed that children with the following characteristics were less likely to have symptoms of depression: one or both parents having >9 years of education (OR, 0.78, 95% CI: 0.63–0.96, *p* = 0.02), those with longer sleep duration (OR, 0.90 95% CI: 0.81–0.97, *p* = 0.03) and study time (OR, per hour 0.82, 95% CI: 0.74–0.91, p = 0.03). Children with higher recreational screen time were significantly more likely (OR per additional hour 1.19, 95% CI: 1.08–1.32, *p* = 0.001) to have depression symptoms. Being a left-behind child also marginally increased the risk of depression (OR,1.26, 95% CI: 0.99–1.59, *p* = 0.057) (Table 3).

**Table 3:** Factors associated with symptoms of depression, anxiety, and stress among rural students in China.

Variables	Symptoms of depression (DASS-D > 9)		Symptoms of anxiety (DASS-A > 7)		Symptoms of stress (DASS-S > 14)	
	Bivariate model, beta value, 95% CI	Multivariate model, beta value, 95% CI	Bivariate model, beta value, 95% CI	Multivariate model, beta value, 95% CI	Bivariate model, beta value, 95% CI	Multivariate model, beta value, 95% CI
Visual impairment	1.009 (0.79, 1.29)		1.03 (0.83, 1.29)		1.18 (0.90, 1.39)	
Glasses ownership	0.94 (0.76, 1.19)		0.97 (0.79, 1.19)		0.85 (0.69, 1.04)*	0.86 (0.70, 1.06)
Female gender	0.97 (0.80, 1.18)		0.95 (0.80, 1.13)		1.11 (0.93, 1.32)	
Age	1.13 (1.01, 1.26)*	1.09 (0.97, 1.22)	1.05 (0.95, 1.16)		1.13 (1.02, 1.25)*	1.12 (1.004, 1.24)**
Grade 8 (vs. 7)	1.02 (0.84, 1.24)		1.03 (0.86, 1.23)		1.07 (0.89, 1.27)	
Boarding at school	1.01 (0.83, 1.24)		1.07 (0.90, 1.29)		1.10 (0.92, 1.32)	
Han (vs. Hui) ethnicity	0.97 (0.77, 1.23)		0.97 (0.79, 1.20)		0.81 (0.66, 1.005)*	0.81 (0.64, 1.02)
Number of siblings	0.97 (0.89, 1.05)		1.03 (0.96, 1.11)		1.10 (1.02, 1.19)*	1.10 (1.01, 1.19)**
Family wealth						
Poorest tercile	Reference		Reference		Reference	
Middle tercile	0.89 (0.69, 1.13)*	0.90 (0.69, 1.17)	1.06 (0.85, 1.33)		1.08 (0.87, 1.36)	
Highest tercile	0.81 (0.64, 1.01)*	0.91(0.71, 1.17)	0.86 (0.70, 1.06)		0.93 (0.76, 1.14)	
One or both parents with >9 years of education	0.76(0.62, 0.92)*	0.78 (0.63, 0.96)**	0.76 (0.63, 0.91)*	0.80(0.66, 0.96)**	0.89 (0.74, 1.07)	
Left-behind child	1.21(0.96, 1.52)*	1.26 (0.99, 1.59)	1.18 (0.98, 1.40)*	1.26(1.04, 1.54)^**^	1.10 (0.90, 1.33)	
Parents not married	1.23(0.90, 1.68)*	1.43 (0.99, 2.05)	1.10 (0.83, 1.49)		1.06 (0.79, 1.43)	
Study time (hours)	0.82(0.74, 0.91)*	0.82 (0.74, 0.91)^**^	0.96 (0.88, 1.04)*	0.95(0.87, 1.03)	0.97 (0.89, 1.05)	
Recreational screen time (hours)	1.17(1.06, 1.29)*	1.19 (1.08, 1.32)^**^	1.14 (1.03, 1.25)*	1.15(1.04, 1.27)^**^	1.16 (1.05, 1.29)*	1.15 (1.04, 1.28)^**^
Sleep time (hours)	0.95(0.87, 1.03)*	0.90 (0.81, 0.97)	1.00 (0.88, 1.14)		0.94 (0.87, 1.02)*	0.92 (0.85, 0.99)
Outdoor activity time (hours)	0.95(0.83, 1.10)		1.02 (0.90, 1.16)		1.02 (0.89, 1.16)	

*p < 0.2 in the bivariate analysis were exported to the multivariable regression model. **p < 0.05.

Children with one or both parents having >9 years of education were significantly less likely (OR, 0.80, 95% CI: 0.66–0.96, *p* = 0.02) to have anxiety symptoms. Children with higher recreational screen time (OR per additional hour 1.15, 95% CI: 1.04–1.27, *p* = 0.004) and left-behind children were significantly more likely (OR, 1.26, 95% CI: 1.04–1.54, *p* = 0.016) to suffer from anxiety symptoms (Table 3).

Children with more siblings (OR per additional sibling 1.10, 95% CI: 1.01–1.19, *p* = 0.002), who engaged in higher recreational screen time (OR per hour, 1.15, 95% CI: 1.04–1.28, *p* = 0.009), and who were older (OR per year, 1.12, 95% CI: 1.004–1.24, *p* = 0.04) were significantly more likely to experience symptoms of stress. Children with longer sleep duration per day were significantly less likely (OR, 0.92, 95% CI: 0.85–0.99, *p* = 0.002) to have symptoms of stress. Ethnicity was not associated with any of the three mental health outcomes in regression models (Table 3).

## Discussion

The current study provides insight into the mental health problems of secondary school children living in Northwestern rural China during COVID-19. Approximately a quarter of children experienced symptoms of depression and stress, and close to half of the sample experienced symptoms of anxiety. Modifiable factors, such as longer recreational screen time and shorter sleeping time, and sociodemographic factors, such as having parents with fewer years of education, being left-behind children, being older and having more siblings, were associated with poorer mental health.

Studies in Chile (33), Bangladesh (34), and Palestine (35) using the same mental health questionnaire as our study found a much higher prevalence of symptoms of depression, anxiety, and stress. However, all these studies collected data through online questionnaires, which will make the study participants non-representative. The prevalence of depression and anxiety in the current study was higher than reported in a systematic review, which found that the pooled prevalence of depression and anxiety symptoms among Chinese children during COVID-19 were 22 and 25%, respectively (36). Even though Ningxia was one of the less severely impacted areas of China (37), our study suggests that children may still have been affected by negative experiences related to fear of infection, lack of personal contact with friends, increased screen time and reduced outdoor time during the pandemic, and thus, experienced more depression and anxiety symptoms.

Measures such as school closures and the transition to online studies contributed to the increased use of screen-based media for education and recreation (38–40). Screen-based sedentary behaviors have been recognized as a significant contributor to adverse outcomes such as mental health problems (41), obesity (42), and myopia (43). The average recreational screen time reported in the current study was less than an hour per day, much less than previous studies conducted in other parts of China, and yet had a significant association with increased mental health symptoms (44). According to guidelines issued by the Health Department of China during the COVID-19 pandemic, secondary school children should be exposed to no more than 4 h of online study and 1 h of recreational screen time per day (45). Our findings may help inform stakeholders considering interventions such as developing a family media plan (46) to mitigate the potential risks to children’s mental health of screen use.

We identified shorter study time and sleep duration as two potentially modifiable risk factors associated with poorer mental health. Given that many intermediate school children in rural China aspire to continue to academic high schools, which requires a high entrance examination score, spending less time studying might have caused anxiety over educational prospects. Many Chinese adolescents were already not meeting the recommended sleep time of 8–10 h per night before the pandemic (47). Children with excessive screen time leading to shorter sleep duration (48), going to bed late and taking a long time to fall asleep during the pandemic are all associated with adverse physical and mental health outcomes (49, 50). Hence, public health messages regarding the importance of healthy sleep schedules should be disseminated to children and parents.

Previous reviews, including studies of Christian populations, have indicated that religious involvement is correlated with better mental health (51). With its Islamic practices, the Hui ethnicity might be hypothesized to protect against mental health problems. However, economic development and education in recent years in China have led to reduced emphasis on religious practices among some Hui (52). This trend of cultural integration may have contributed to the lack of significant differences in the prevalence of the three mental health symptoms between Hui and Han children.

Our findings suggest that vision impairment was not associated with symptoms of depression and anxiety, which is inconsistent with a recent systematic review (15). The daily outdoor time of the children included in this study was far below the 2 h per day suggested by the National Health Commission of the People’s Republic of China (45). Furthermore, despite studies showing that outdoor activities can relieve anxiety and stress (53, 54), we did not find the same associations. Hence, we hypothesize that the disruption in lifestyle habits during the pandemic had greater detrimental effects on mental health than vision impairment. During the COVID-19 pandemic, learning activities in secondary schools alternated from face-to-face to online if there were new COVID cases. In the face-to-face context, children with vision impairment might have experienced stress due to difficulty in seeing the material on a blackboard, and the ensuing academic pressures. In addition, during COVID-19, the transition of traditional class to online one was a challenge to students due to technology-related issues (55), and lack of interaction with teachers and fellow students (56).

Children from socioeconomically disadvantaged families and those left behind at home were more likely to experience mental health symptoms than their peers without these issues (57, 58). Left-behind children are usually cared for by their grandparents, and inadequate parental care may impact children’s physical and mental health (58). Additionally, left-behind children’s families may have suffered reductions in income due to the pandemic. Amidst the lockdown in 2020, around 25 million rural migrants in China found themselves either unemployed or in a state of anticipation, awaiting the opportunity to return to work (59). Many migrant parents fail to maintain a strong and healthy parent–child relationship (60). While the lockdown created an opportunity for parents and left-behind children to spend more time together, repeating separations and reunions with caregivers is stressful. This may have disrupted left-behind children’s attachment behaviors during the pandemic (61).

Interestingly, children with more siblings were more likely to report stress on the DASS-21 than those with fewer siblings or no siblings. This is consistent with a previous study that found that children with siblings were more likely to develop symptoms of anxiety and depression compared to only-children during COVID-19 (62). Studies before COVID-19 found better mental health status in children with siblings compared to children without siblings (63, 64). In addition to effecting health, COVID-19 severely decreased employment and income (59, 65). Underprivileged students may not have access to resources such as the internet, which are essential for their learning (66). We hypothesize that, families with fewer children may have had fewer economic burdens and could provide more attention and support to children, but these attitudes and beliefs could also be contextualized in decades-long single-child family restrictions.

Strengths of the current study include the focus on China’s relatively less-studied rural west, particularly the Hui ethnic group, about whose mental health epidemiology relatively little is known. A large sample of children was recruited, with high rates of participation, reducing risk of bias, and a well-recognized tool, validated in China, was used to assess mental health symptoms.

Several limitations of this study should also be noted. Firstly, the sample was taken from 3 counties only and might not represent children throughout Ningxia Hui Autonomous Region. Secondly, recall and reporting bias might have occurred, with variables such as sleep time and recreational screen time being self-reported. In addition, the individual history and family history of psychiatric disorders were not collected, which might confound the association between mental health symptoms and risk factors. Finally, as this study was cross-sectional, we could not establish a causal relationship between mental health and risk factors. Despite the limitations, this is the first study investigating the mental health of Hui children and identified several modifiable risk factors of potential interest to policymakers.

## Conclusion

Our results showed that a considerable proportion of these children in Northwestern China experienced depression, anxiety, and stress symptoms during the COVID-19 pandemic. Healthcare planners in China should consider interventions such as reducing recreational screen time, ensuring sufficient sleep, and timely detection of mental health symptoms among socioeconomically disadvantaged groups.

## Data availability statement

The dataset of this article will be uploaded to Queen’s University Belfast Research Portal and further inquiries can be directed to the corresponding author.

## Ethics statement

The studies involving humans were approved by the Stanford University and Queen’s University Belfast. The studies were conducted in accordance with the local legislation and institutional requirements. Written informed consent for participation in this study was provided by the participants’ legal guardians/next of kin.

## Author contributions

DL: Conceptualization, Formal analysis, Methodology, Software, Writing – original draft, Writing – review & editing. VC: Conceptualization, Formal analysis, Methodology, Writing – review & editing, Supervision. HW: Conceptualization, Methodology, Writing – review & editing, Project administration, Validation. HZ: Methodology, Project administration, Writing – review & editing. GV: Methodology, Writing – review & editing, Formal analysis. NW: Writing – review & editing, Conceptualization. BX: Writing – review & editing, Project administration. MS: Writing – review & editing, Conceptualization, Methodology. XS: Conceptualization, Methodology, Writing – review & editing. GM: Conceptualization, Writing – review & editing, Funding acquisition, Resources. MB: Conceptualization, Funding acquisition, Writing – review & editing. SM: Writing – review & editing, Methodology. SR: Methodology, Writing – review & editing, Conceptualization, Funding acquisition. NC: Conceptualization, Funding acquisition, Writing – review & editing, Project administration, Resources, Supervision.
